# Fine-mapping of intracranial aneurysm susceptibility based on a genome-wide association study

**DOI:** 10.1038/s41598-022-06755-x

**Published:** 2022-02-17

**Authors:** Eun Pyo Hong, Dong Hyuk Youn, Bong Jun Kim, Jun Hyong Ahn, Jeong Jin Park, Jong Kook Rhim, Heung Cheol Kim, Gyojun Hwang, Hong Jun Jeon, Jin Pyeong Jeon

**Affiliations:** 1 Institute of New Frontier Research, Hallym University College of Medicine, Chuncheon, Republic of Korea; 2 Department of Neurosurgery, Gangwon National University College of Medicine, Chuncheon, Gangwon-do Republic of Korea; 3 Department of Neurology, Konkuk University Medical Center, Seoul, Republic of Korea; 4 Department of Neurosurgery, Jeju National University College of Medicine, Jeju, Republic of Korea; 5 Department of Radiology, Hallym University College of Medicine, Chuncheon, Gangwon-do Republic of Korea; 6 Department of Neurosurgery, DMC Bundang Jesaeng Hospital, Seongnam, Gyeonggi-do Republic of Korea; 7 Department of Neurosurgery, Hallym University College of Medicine, 77 Sakju-ro, Chuncheon, Gangwon-do 24253 Republic of Korea

**Keywords:** Computational biology and bioinformatics, Genetics, Neuroscience, Biomarkers, Diseases, Neurology, Risk factors

## Abstract

In addition to conventional genome-wide association studies (GWAS), a fine-mapping analysis is increasingly used to identify the genetic function of variants associated with disease susceptibilities. Here, we used a fine-mapping approach to evaluate candidate variants based on a previous GWAS involving patients with intracranial aneurysm (IA). A fine-mapping analysis was conducted based on the chromosomal data provided by a GWAS of 250 patients diagnosed with IA and 296 controls using posterior inclusion probability (PIP) and log10 transformed Bayes factor (log10BF). The narrow sense of heritability (*h*^2^) explained by each candidate variant was estimated. Subsequent gene expression and functional network analyses of candidate genes were used to calculate transcripts per million (TPM) values. Twenty single-nucleotide polymorphisms (SNPs) surpassed a genome-wide significance threshold for creditable evidence (log10BF > 6.1). Among them, four SNPs, rs75822236 (*GBA*; log10BF = 15.06), rs112859779 (*TCF24*; log10BF = 12.12), rs79134766 (*OLFML2A*; log10BF = 14.92), and rs371331393 (*ARHGAP32*; log10BF = 20.88) showed a completed PIP value in each chromosomal region, suggesting a higher probability of functional candidate variants associated with IA. On the contrary, these associations were not shown clearly under different replication sets. Our fine-mapping analysis suggested that four functional candidate variants of *GBA*, *TCF24*, *OLFML2A*, and *ARHGAP32* were linked to IA susceptibility and pathogenesis. However, this approach could not completely replace replication sets based on large-scale data. Thus, caution is required when interpreting results of fine-mapping analysis.

## Introduction

Intracranial aneurysm (IA) refers to an abnormal focal dilatation of a cerebral artery due to a weakening of the intima of a blood vessel wall. The prevalence of IA in the general adult population has been reported to be nearly 6%^1^. The incidence of unruptured intracranial aneurysms (UIAs) was 15.6 per 100,000 persons^2^. The asymptomatic UIA can rupture suddenly resulting in subarachnoid hemorrhage (SAH), which is associated with a higher mortality rate exceeding 50% within one month after ictus^3,4^. IA is a complex disease involving an interaction between clinical and genetic factors underlying its formation and growth^2^. Important clinical risk factors for IA include female gender, hypertension and smoking. The risk of rupture is increased when the aneurysm is located between arterial branches or in the vertebrobasilar region, in addition to larger size at diagnosis, and the presence of a bleb or daughter sac^5–7^. Genetic studies have been performed to identify genes associated with IA via linkage analysis and single nucleotide polymorphisms (SNPs) of known candidate genes, strongly correlated genes or genome-wide association studies (GWAS) for screening multiple candidate genes. In particular, GWAS revealed large-scale genetic associations, which were primarily correlated with traits and diseases. GWAS technically compares allele frequencies in SNPs between cases and controls. However, complex traits of IA are not entirely attributed to a single gene, but are caused by the influence of multiple genes^8,9^. Gene–gene and gene–environment interactions also affect the traits and diseases. Given the inherent features of the GWAS, genetic markers included in the same linkage disequilibrium exhibit similar correlation. Accordingly, even if a candidate gene is identified via GWAS, it could merely suggest a statistically significant difference rather than represent an etiological factor. Further, the precise location of the causative gene may differ in the same linkage disequilibrium (LD) block. Thus, it is important to reduce the errors via additional data processing to identify false-positive results obtained in GWAS.

Fine-mapping is one of the post-GWAS analyses used to narrow potential candidate variants directly affecting the trait^10^. This approach can be used to identify the regions associated with possible susceptibility based on the population of structures with LD^9^. It provides a complex correlation between the candidate variants and the disease development using computational data without in vivo and in vitro molecular biology studies^10,11^. Sekar et al.^11^ showed that structurally diverse alleles of the complement component 4 genes contribute to schizophrenia via excessive complement activity, resulting in reduced numbers of synapses. Consequently, it can be used to assess the functional role of the risk allele, which is a challenge to investigate based on molecular mechanisms, despite the strong genetic association. Fine-mapping of complex traits has been increasingly performed in many diseases, especially cancer and stroke, but has yet to be reported in IA. Here, for the first time, we performed a fine-mapping analysis based on previous GWAS data sets to identify the candidate variants in an effort to identify the precise genetic variants associated with IA in a Korean adult cohort. We also performed a functional gene set enrichment analysis using the optimized candidate sets to analyze the biological relationship between candidate genes and IA.

## Materials and methods

### GWAS-based summary statistics

The analysis was based on the summary statistics provided by the previous IA GWAS. In brief, the study included 250 adult patients with saccular aneurysm and 296 controls between March 2015 and December 2020^12,13^. The Axiom^TH^ Asian Precision Medicine Research Array (APMRA) (Thermo Fisher Scientific, MA, USA) were used for genotyping of the study subjects. High-quality plates were defined by a plate pass rate higher than 95% for samples. The average call rate of passing samples was greater than 99%. A total of 512,575 SNPs passed the quality control including genotyping call rate of 95% or higher, minor allele frequency (MAF) at least 1%, and Hardy–Weinberg equilibrium (HWE) with a *p* value ≥ 1 × 10^–6^^12^. GWAS-based summary statistics included allele types, MAF, and effect sizes. All methods were carried out in accordance with relevant guidelines and regulations. This study protocols including all subjects providing written informed consents has been approved by The Institutional Review Board of the Hallym University Chuncheon Sacred Heart Hospital (No. 2016-3 and 2019-06-006-012).

### Statistical analysis

We performed a fine-mapping study to identify the role of candidate combinations in the susceptibility to IA using FINEMAP v.1.3.1^12,14^. Odds ratios (ORs) of individual SNPs were converted using the natural log-transformed formula (lnOR). Causality of each SNP or configuration was assessed using effect sizes, posterior inclusion probabilities (PIPs), and narrow sense of heritability (*h*^2^), which was explained by candidate SNPs. The log10-transformed Bayes factors (log10BF) for the individual SNPs and configurations were estimated via FINEMAP analysis. A log10BF value of above 2 was considered a creditable threshold in the FINEMAP. However, a log10BF value of greater than 6.1 suggested significant genome-wide evidence in GWAS. The fine-mapping approach requires the estimates of SNP correlations, and therefore LD matrices between SNPs were generated by PLINK v1.9 (https://www.cog-genomics.org/plink/)^15^. All the fine-mapping tests were conducted with individual chromosomes (chr1-22) due to the LD-based mapping procedure. Manhattan and regional association plots of fine-mapping results were obtained using the package of “*qqman*” in R v3.6.1 (https://cran.r-project.org/web/packages/qqman) and LocusZoom v1.3 written in the modified Python and R scripts^16^. Regional annotations and functional impact of SNPs were described using the ANNOVAR program (http://www.openbioinformatics.org/annovar/)^17^.

### Gene expression and functional network analyses

The expressions of candidate genes was evaluated in human blood, brain-specific tissues, or cells using the Geotype-Tissue Expression (GTEx) Portal (https://gtexportal.org/home/)^18^. Transcripts per million (TPM) values of a total of 56,200 genes were calculated in 13 brain tissues, 4 blood vessels (3 arterial tissues and 1 cell line of EBV-transformed lymphocytes), and a whole blood cell. Subsequent gene functional network analysis was conducted using the GeneMANIA program (https://genemania.org/)^19^.

### Subsequent validation of 29 SNPs under different replication stages

Targeted loci of 29 SNPs suggested by fine-mapping analysis based on initial GWAS were tested using the following two independent sets: (1) stage 2, independent subjects of 50 patients with IA and 46 controls from two Hallym University hospitals, Chuncheon and Gangdong Sacred Heart Hospitals (IA group: 24 males and 26 females aged 28–80 years; control group: 22 males and 24 females, aged 19–72 years); and (2) stage 3, the same independent set of 50 hospital-enrolled patients in stage 2 and another 575 healthy controls without underlying diseases. Controls were extracted from 8105 subjects of the Rural and Mid-size City cohort of the Korean Genome Epidemiology Study. We further accounted for a stringent significance threshold of 0.001724 according to multiple testing (0.05/29 SNPs).

## Results

### Fine-mapping analyses

**Figure 1:**
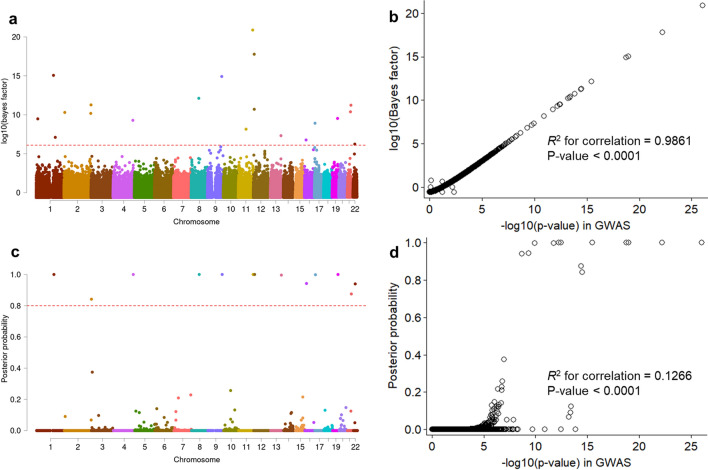
(**a**,**c**) Manhattan plots show log10-transformed Bayes factors (log10BF) and posterior inclusion probability (PIP) of variant causality estimations based on the summary statistics of a genome-wide association study (GWAS) of intracranial aneurysm (IA). (**b**,**d)** Plots compare the significance of IA GWAS, log10BF, and PIP. A red dash line indicates a genome-wide significance and a strong PIP of IA formation in the panels (**a**) and (c), respectively (log10BF = 6.1 and PIP = 0.8). R-square (*R*^2^) indicates the correlation between IA GWA p-value (− log10 transformed) and log10BF in panel (**b**) and PIP in panel (**d**): *p*-value for *R*^2^.

Study workflow and summarized outcomes are shown in Supplementary Fig. S1. Fine mapping analyses entailed multiple SNP combinations by each autosome chromosome. A total of 20 candidate SNPs surpassed a genome-wide significance threshold for creditable evidence (log10BF > 6.1) (Fig. 1a). The log10BF values were strongly correlated with GWAS-based p-values (*R*^2^ for correlation = 0.9861, p < 0.0001) (Fig. 1b). Among them, 13 SNPs showed a high PIP in each chromosomal region (PIP > 0.8) (Fig. 1c). The PIPs were slightly correlated with GWAS-driven *p*-values (*R*^2^ for correlation = 0.0974, *p* < 0.0001) (Fig. 1d). Six out of 13 SNPs were located on exonic regions (Table 1). The rs371331393 (Q1932X), one SNP of *ARHGAP32* (11q24.3), exhibited a stop-gain function and was most significantly associated with increased IA risk (lnOR = 3.77, log10BF = 20.88). This variant showed the highest heritability (*h*^2^ = 0.143). Three SNPs including rs75822236 (R535H, *GBA*; log10BF = 15.06), rs112859779 (G141S, *TCF24*; log10BF = 12.12), and rs79134766 (A208T, *OLFML2A*; log10BF = 14.92) were associated with amino acid substitutions, which may contribute to possible DNA sequence damage and increased association with IA. Remaining 16 IA-predicting candidate loci (6 intergenic, 8 intronic, and 2 synonymous SNPs; log10BF > 6.1 and *p* < 5 × 10^–8^) were not accompanied by any neighbor promotor or functional variations such as UTR, missense, and nonsense SNPs that showed both moderate to strong LD (0.5 < *r*^2^ < 1) and marginally significant associations (*p* < 0.0001, data not shown). This implicated that these SNPs could be commonly predictable false positive outcomes or potentially related to other mechanisms such as alternative splicing and gene fusions, but not associated with IA. Eighteen SNPs, including *PRDM2, FMO4*, and *RNF144A*, which exceeded genome-wide significance level in GWAS (*p* < 5 × 10^–8^), were associated with low causality to IA formation (1.89 × 10^–11^ < PIP < 0.13, log10BF > 5) (Supplementary Table S1). In summary, fine mapping annotated that *ARHGAP, GBA, TCF24,* and *OLFML2A* could be potentially candidate genes contributing to DNA damaging. However, functional mutations around 16 other genes including intergenic, intronic, non-coding RNA, and synonymous loci were not discovered or they showed insignificant associations in this study (log10BF < 2 or *p* > 0.05, data not shown). Detailed information including SNP allele frequency and HWE *P* value is described in Supplementary Table S1.

**Table 1:** Significant candidate loci identified by fine-mapping after genome-wide association study.

Gene	Chr	Function	SNP	M/m^a^	MAF	PIP^b^	log10BF^b^	*h*^2b^	lnOR	*P* in GWAS	HWE p
*PRDM2*	1p36.21	Intronic	rs61775135	C/A	0.285	2.61E−06	9.48	0.0733	− 1.15	3.59E−13	0.626
*GBA*	1q22	R535H, exon11	rs75822236	C/T	0.166	1.0000	15.06	0.1088	5.08	1.09E−19	1
*FMO4*	1q24.3	F281F, exon8	rs3737926	C/T	0.264	1.10E−08	7.10	0.0577	− 1.01	1.83E−10	0.079
*RNF144A*	2p25.1	Intron	rs6741819	C/T	0.247	0.0905	10.30	0.0785	− 1.38	4.05E−14	0.022
*HDAC4,LOC150935*	2q37.3	Intergenic	rs59626274	C/T	0.248	0.0679	10.18	0.0777	− 1.34	5.78E−14	0.002
*LINC01237*	2q37.3	ncRNA, intron	rs78458145	G/A	0.285	0.8416	11.27	0.0847	− 1.41	3.14E−15	0.007
*SPCS3,VEGFC*	4q34.2	Intergenic	rs17688188	G/A	0.222	0.9999	9.29	0.0718	− 1.31	5.99E−13	0.030
*TCF24*	8q13.1	G141S, exon4	rs112859779	C/T	0.216	1.0000	12.12	0.0898	− 1.69	3.33E−16	2.02E−04
*OLFML2A*	9q33.3	A208T, exon4	rs79134766	G/A	0.219	1.0000	14.92	0.1072	− 1.97	1.70E−19	0.054
*MYEOV,LINC01488*	11q13.3	Intergenic	rs76855873	C/T	0.269	1.89E−13	8.15	0.0642	− 1.11	1.23E−11	0.023
*ARHGAP32*	11q24.3	Q1932X, exon22	rs371331393	G/A	0.171	1.0000	20.88	0.1435	3.77	9.32E−27	1
*CD163L1*	12p13.31	Splicing	rs138525217	C/T	0.161	1.0000	17.77	0.1248	4.33	6.20E−23	1
*SLC2A14*	12p13.31	Intron	rs118107419	C/A	0.262	8.60E−08	10.71	0.0807	− 1.40	1.44E−14	0.012
*CUL4A,LAMP1*	13q34	Intergenic	rs74115822	G/A	0.112	0.9960	7.32	0.0584	1.83	1.12E−10	0.001
*MIR365A,PARN*	16p13.12	Intergenic	rs11646803	C/T	0.376	0.9422	6.77	0.0548	− 0.79	4.76E−10	0.074
*MINK1*	17p13.2	Intron	rs72835045	G/A	0.220	0.9984	8.92	0.0688	− 1.38	1.69E−12	1.16E−06
*NAPA-AS1*	19q13.32	ncRNA, intron	rs55800589	G/C	0.364	1.0000	9.53	0.0726	− 0.96	3.35E−13	2.37E−06
*DSCAM*	21q22.2	Intron	rs727333	C/A	0.257	0.1247	10.38	0.0773	− 1.36	3.69E−14	4.75E−04
*LRRC3*	21q22.3	P63P, exon2	rs116969723	G/A	0.233	0.8753	11.23	0.0827	− 1.45	3.83E−15	0.014
*RFPL2,SLC5A4*	22q12.3	Intergenic	rs117398778	T/C	0.138	0.9397	6.24	0.0506	1.32	2.00E−09	1.56E−05

*GWAS* genome-wide association study, *log10BF* log10 transformed Bayes factor, *lnOR* natural log-transformed odds ratio, *PIP* posterior inclusion probability, *MAF* minor allele frequency.

^a^M/m indicates major/minor allele type, respectively.

^b^PIP, log10BF, and heritability (*h*^2^) of individual variants were estimated via FINEMAP program to identify possible susceptibility to intracranial aneurysm (IA).

^c^lnOR and *P*-value were estimated by IA GWAS.

### Gene expression and functional network analyses

**Figure 2:**
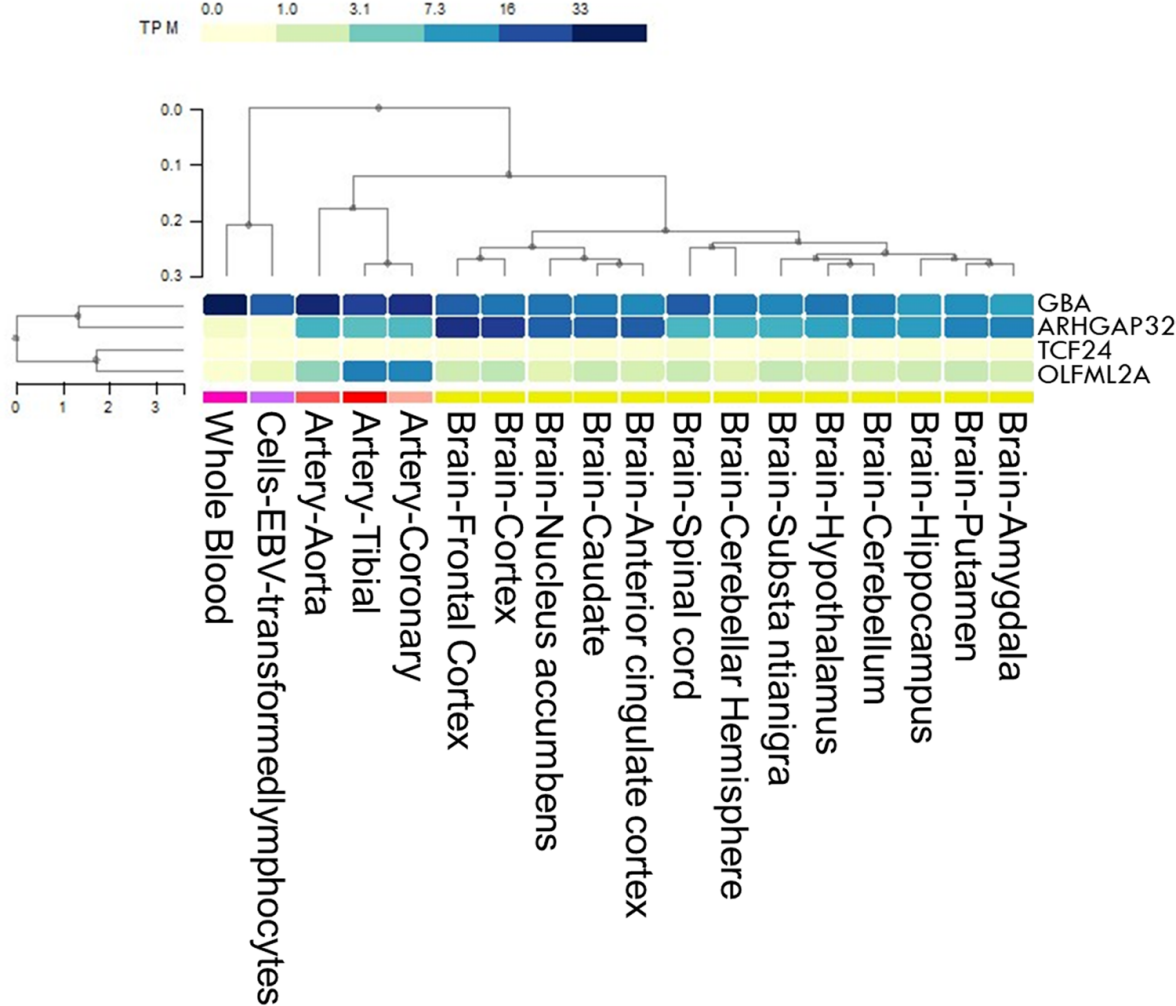
A heatmap of multiple gene expression involving *GBA*, *TCF24*, *OLFML2A*, and *ARHGAP32* in human cells and tissues including artery, brain, and whole blood is presented. Gene expression was estimated as transcripts per million (TPM). Genes and types of cells or tissues were ordered via agglomerative hierarchical clustering. An interactive heatmap specifically designed for rendering expression data was drawn by the GTEx Expression Map tool to report and summarize multi-gene and multi-tissue expressions (https://gtexportal.org/home/multiGeneQueryPage).

**Figure 3:**
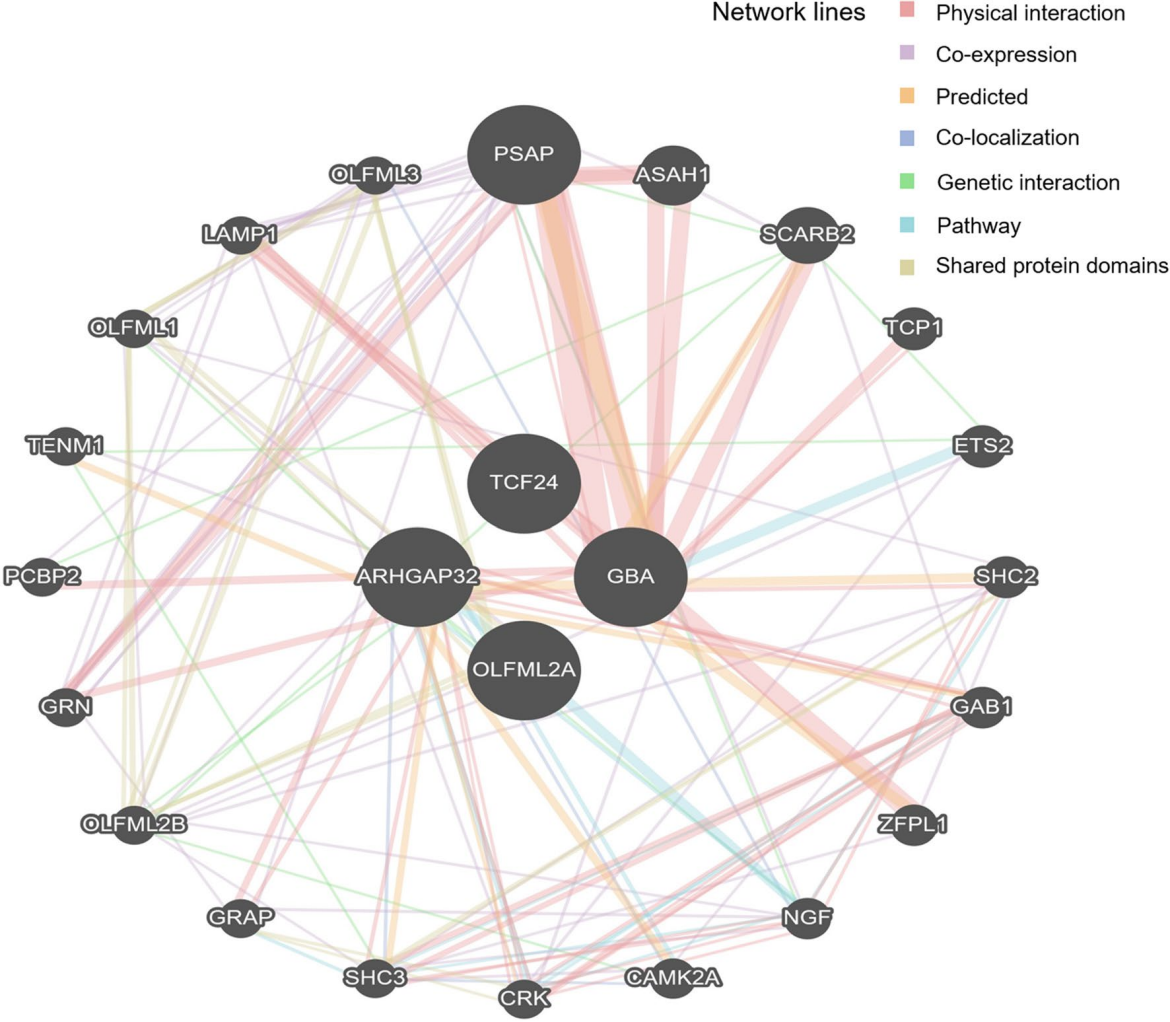
Susceptibility to intracranial aneurysm (*Homo sapiens*) based on multiple protein interactions between proteins coded by four candidate hub genes including *GBA*, *TCF24*, *OLFML2A*, and *ARHGAP32.* The network included neighboring genes correlated with four hub genes. The width of individual lines indicates the intensity of the interaction between proteins. The colors in each line indicate multiple functions including physical interaction, co-expression, prediction, co-localization, genetic interaction, pathways, and shared protein domains. The multiple protein interaction map was drawn by using the GeneMANIA program (https://genemania.org/).

Gene expression and functional network analyses were performed using the four candidate genes (Fig. 2 and Supplementary Table S2). *GBA* was broadly enriched in all tissues and cell lines (6 < TPM < 34). In particular, it was highly expressed in the whole blood (TPM = 33.13). Conversely, *TCF24* was rarely expressed in all tissues and cells (TPM < 0.15). Expression in *OLFML2A* was moderate in all arteries (TPM = 2.37 to 9.54). *ARHGAP32* was rarely enriched in EBV-transformed lymphocytes and whole blood (TPM = 0.0667 and 0.2274, respectively), while it was enriched in the brain and blood vessels (TPM, between 3 and 24). No direct interaction was observed between the four candidate genes (Fig. 3). However, these genes constitute a hub network interacting with neighbor genes, especially *PSAP*, *SCARB2*, and *ASAH1* (Fig. 3).

### Subsequent validation of 29 SNPs after fine-mapping

Out of a total 29 SNPs that revealed by fine-mapping analysis based on initial GWAS, only five SNPs such as rs79461840, rs4979583, rs7964241, rs2440154, and rs117398778 showed a statistical significance at independent stage 2 (0.002 < *p* < 0.05). Five SNPs such as rs75822236, 9_127561723. rs371331393, rs138525217, and rs55800589 could not be analyzed in fully at this replication stage because minor alleles of these SNPs show no frequency in the control group. Another replication stage 3 showed that only eight SNPs were matched with the GWAS-driven fine mapping analysis. Among them, two SNPs such as rs56942085 (*LINGO2*) and rs2440154 (*SLC47A1*) were closely associated with IA (*p* = 0.03301 and 0.01104, respectively). During replication studies, only one SNP rs2440154 showed a constant association with IA (*p* < 0.05), but did not surpass an adjusted *p*-value less than 0.05 after multiple testing correction (*p* < 0.001724) (Supplementary Table S3).

## Discussion

This study performed additional fine mapping analysis based on the original GWAS and found four candidate variations of *GBA*, *TCF24*, *OLFML2A*, and *ARHGAP32* genes potentially linked to IA in Koreans. We speculated that these genetic variants may cause dysfunctional immune response and inflammation in DNA sequences damaged by amino acid substitution or gain- or loss-of-function mutations, which affects the IA formation. The “T” allele of rs75822236 located in the exonic region of *GBA* increased the risk of IA via previous GWAS^12^. In addition, a fine-mapping analysis revealed a higher level of log10BF (15.06) and PIP (1.0), suggesting that this variant was a true positive for IA, although there might be concerns about its reliability due to the small sample size of the original GWAS datasets. The role of *GBA* was mainly investigated in Parkinson’s disease (PD) or Gaucher disease (GD), which is a recessive lysosomal storage disorder, and barely investigated in IA. Mata et al.^20^ reported that *GBA* mutations and E326K carrier were related to impaired working memory and executive function in patients with PD. In GD, null or severe homozygous mutations of *GBA* showed little or no human glucocerebrosidase activity^21^. These findings suggested differences in phenotype due to the various *GBA* mutations. Kleinloog et al.^22^ reported enrichment of the lysosomal pathway in ruptured IA compared with UIA based on RNA sequencing analysis of aneurysm wall. Although the lysosomal pathway does not reflect an acute reaction to IA rupture^22^, it is likely that it is induced by inflammation after bleeding.

*OLFML2A* and *TCF24* showed a protective effect against IA formation with log10BF levels greater than 12 and a PIP of 1.0. However, the relationship between these two genes and IA is still unclear, even though it has been implicated in cardiovascular diseases. Conversely, *ARHGAP32* significantly increased the risk of IA with a log10BF of 20.88 and a PIP of 1.0. *ARHGAP32* refers to Rho GTPase-activating protein 32 and mediates *N*-methyl d-aspartate receptor signaling^12^. The role of *ARHGAP32* has been mainly investigated in the regulation of blood pressure. Rho-specific GTPase-activating protein *GRAF3* was highly expressed in smooth muscle cells (SMCs) and regulated blood pressure control by inhibiting the contractility of RhoA-mediated SMC^23^. GRAF3-deficient mice also showed increased blood pressure in response to angiotensin II and endothelin 1^24^. In actual clinical practice, many patients manifest both IA and hypertension. Inci et al.^25^ reported that the rate of pre-existing hypertension was 43.5% in patients with IA, which was higher than 24.4% in the normal population. Hypertension may contribute to degeneration of the internal elastic lamina, weakening of the vessel wall, and IA formation^25^.

Nevertheless, it is unclear whether the role of *ARHGAP32* in IA is mediated indirectly via chronic hypertension or directly via change in vascular tone.

Functional network analyses showed that *PSAP* was an important gene in the development of IA. The role of *PSAP* gene was rarely investigated in IA and was mainly studied in PD. Oji et al.^26^ reported that two SNPs of rs4747203 and rs885828, the intronic regions of the *PSAP* saposin D domain were linked to PD. *PSAP* mutation can also result in dopaminergic neurodegeneration and motor decline in mice. Although we did not include patients with PD, a fine-mapping analysis revealed that PD-related genes such as *GBA* and *PSAP* may contribute to IA. Lysosomal dysfunction and the resulting lysosomal storage disorder can contribute causally to PD. Putative damaging variants in at least one gene associated with lysosomal storage disorder were observed in most PD patients^27^. However, lysosomal dysfunction can also be observed in the arterial wall. Lysosomal changes in the vascular SMCs were attributed to the accumulation of excessive substrate levels in the lysosomes of a primate model of atherosclerosis and hypertension^28^. The excessive sterol accumulation in lysosomes can disrupt the lysosomal function^29^. Therefore, in this case, it is possible that lysosomal dysfunction may directly affect IA formation or may contribute to IA via atherosclerosis.

Hokari et al.^30^ reported that atherosclerotic factors strongly increased the risk of middle cerebral artery aneurysm compared with paraclinoid aneurysm. After securing aneurysm, consistent statin therapy was significantly correlated with better prognosis^31^. Wu et al.^32^ demonstrated that the autophagy–lysosomal pathway, which entails self‐digestion of dysfunctional intracellular components by lysosomal enzymes, was an important pro-survival mechanism after SAH. However, the study investigated the role of the lysosome after SAH development, but not in IA development itself. Thus, additional studies are needed to investigate the role of candidate variants in lysosomal dysfunction resulting in IA formation via abnormal ECM remodeling in response to hemodynamic stress.

Large-scale GWAS and meta-analyses have been widely used to identify and validate common or novel susceptible gene variants in various medical diseases over the past decade. However, given the overall genotype–phenotype analyses, disease-modifying functional mutations and direct biological relevance to disease have yet to be elucidated completely^33^. In addition, the heritability of a specific trait cannot be fully explained by common SNPs of intronic or intergenic regions via GWAS because GWAS analysis was designed to identify common variants with low and modest effect size, which contribute to disease. Accordingly, even if a large number of susceptible loci were identified, a few cases showed their replication in an independent cohort. Thus, few disease-associated variants have been demonstrated in functional in vitro studies or used in treatment^34^. To overcome these limitations, an updated fine-mapping analysis was performed to identify the candidate variants associated with complex human diseases and as a cost-effective genotyping strategy^9^. To date, many studies underscored the need for ‘feature selection’ to identify relevant “variables” using parametric or non-parametric models. However, feature selection is not a simple challenge and requires substantial genetic investigations. It is important to identify the driver mutations linked to treatment of complex human diseases. The selection of genetic variants from GWAS is uncertain given the strongly correlated SNPs corresponding to a pairwise LD structure at the population level. A fine-mapping analysis facilitates the identification of creditable genetic variants to refine the selection bias such as false-positive variants based on the initial GWAS and to improve the findings of molecular functional studies^9^. Here, we performed a fine-mapping analysis based on the results of previous IA GWAS using the statistical method developed by Benner et al.^14^ Our findings may enable the identification of candidate variants via a pairwise LD structure and exclude potential false positives via statistically significant fine-mapping analysis of transformed GWAS results. Therefore, these analytical methods may enable the selection of functional candidate variants based on the molecular mechanisms associated with IA formation.

Our fine-mapping identified the most likely variant causality among candidate SNPs included in the analysis; however, the actual disease-associated variant might be nearby or highly located at another locus tagged by haplotype structures accompanied by ‘a LD-tower’ in most GWAS. The caution is required to interpret our results through more cross validations, though this is the first fine-mapping analysis based on results of a GWAS of IA to reduce IA-associated variants with false positive. Fine-mapping analysis is inevitably affected by the original datasets. Accordingly, small sample size of the GWAS data without replication study is a concern. In particular, the effect size for the identified SNPs was extremely large. Moreover, the identified significant SNPs were not accompanied by correlated SNP via LD^12^. Since these issues are most likely due to the small sample size, a study based on a large number of patients is needed to address these issues. However, we cannot perform the fine-mapping analysis further based on larger patient data than the current data for two reasons. First, we performed the fine-mapping analysis by a real linkage disequilibrium (LD) based on a real genotype dataset.

Although recently large-scale GWAS meta-analysis results have been published^35^, we cannot obtain a real original dataset or a whole summary statistics and genotype of studies included. Second, we genotyped samples using the APMRA for the first stage GWAS. Accordingly, we identified several novel SNPs that were not found in other GWASs based on Caucasian-based chip array. However, paradoxically, this can be a limitation when comparing data results of other papers. Actually, we analyzed 17 SNPs which showed association with IA in previous genetic studies of IA, including the APMRA used in this study. Of these 17 SNPs, only two SNPs (*BOLL* and *ENDRA*) are associated with IA in Koreans^12^. For these two reasons, we could not perform the fine mapping analysis based on the large dataset. Currently, we are performing IA GWAS as a replication study by analyzing the currently ongoing validation dataset for 50 independent patients with IA and independent controls of hospitals and the Rural and Mid-size City cohort of the Korean Genome Epidemiology Study to validate 29 previous GWA signals showing LD < 0.8^12^. Unfortunately, most candidate SNPs revealed by fine-mapping analysis based on the initial GWAS did not surpass the significance threshold of IA association in replication stages. Therefore, caution is required when interpreting our results because a small number of IA patients were included in our independent replication studies. In addition, some SNPs could not be fully analyzed due to difference in the GWAS panel used.

In summary, fine-mapping analysis robustly identified four functional mutations of candidate genes (*GBA*, *TCF24*, *OLFML2A*, and *ARHGAP32*) associated with IA. Mutations in these genes may play roles in immune and inflammatory systems according to our literature review and functional annotations. At the present, our strategy will offer a good example after GWAS regardless of negative or positive outcomes. For the next step, our findings will provide a milestone of fine-mapping susceptibility to IA development.

## Supplementary Information

Supplementary Information.

## Data Availability

The data that support the findings of this study was submitted as online supplemental material, and further detailed information is available upon request to the corresponding author. All genotype and phenotype resources are managed by “The First Korean Stroke Genetics Association Research” study constructed from the Sacred Heart Hospital Stroke Database.
