# Supplementary material for: Fine-mapping of intracranial aneurysm susceptibility based on a genome-wide association study
Source: Sci Rep. 2022 Feb 17;12:2717. doi: 10.1038/s41598-022-06755-x (PMC8854430; doi:10.1038/s41598-022-06755-x)
Supplement: Supplementary file 1 — Supplementary Information. [file 41598_2022_6755_MOESM1_ESM.pdf]

Fine-Mapping of Intracranial Aneurysm Susceptibility Based on a Genome-Wide Association Study

Eun Pyo Hong[1], Dong Hyuk Youn[1], Bong Jun Kim[1], Jun Hyong Ahn[2], Jeong Jin Park[3], Jong Kook Rhim[4], Heung Cheol Kim[5], Gyojun Hwang[6], Hong Jun Jeon[7] & Jin Pyeong Jeon[7*]

[1]Institute of New Frontier Research, Hallym University College of Medicine, Chuncheon, Republic of Korea.
[2]Department of Neurosurgery, Gangwon National University College of Medicine, Chuncheon, Gangwon-do, Republic of Korea
[3]Department of Neurology, Konkuk Medical Center, Seoul, Republic of Korea
[4]Department of Neurosurgery, Jeju National University College of Medicine, Jeju, Republic of Korea
[5]Department of Radiology, Hallym University College of Medicine, Chuncheon, Gangwon-do, Republic of Korea
[6]Department of Neurosurgery, DMC Bundang Jesaeng Hospital, Seongnam, Gyeonggi-do, Republic of Korea
[7]Department of Neurosurgery, Hallym University College of Medicine, Chuncheon, Gangwon-do, Republic of Korea

SUPPLEMENTAL DATA

Supplementary Figure S1. Study workflow of genome-wide association study, fine-mapping, and further functional research. APMRA, AxiomTH Asian Precision Medicine Research Array; GWA, genome-wide association.

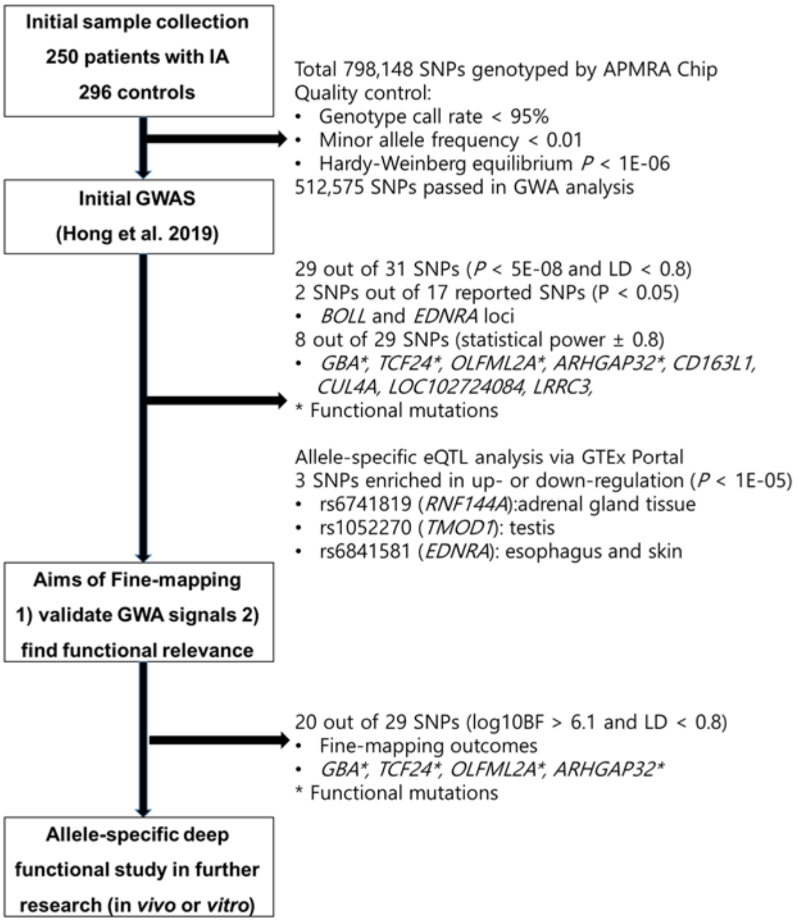

Supplementary Table S1. Comparison of genome-wide association study and FINEMAP results in intracranial aneurysm

Gene	Chr	SNP	Position	Function	Major[a]	Minor[a]	MAF	HWE p	lnOR[b]	P in GWAS[b]	log10BF[c]	PIP[c]	h^{2c}
PRDM2	1p36.21	rs61775135	14148407	intronic	C	A	0.285	0.626	-1.15	3.59E-13	9.48	2.61E-06	0.073
GBA	1q22	rs75822236	155204793	R535H	C	T	0.166	1	5.08	1.09E-19	15.06	1.0000	0.109
FMO4	1q24.3	rs3737926	171303565	F281F	C	T	0.264	0.079	-1.01	1.83E-10	7.10	1.10E-08	0.058
RNF144A	2p25.1	rs6741819	7147973	intronic	C	T	0.247	0.022	-1.38	4.05E-14	10.30	0.0905	0.079
HDAC4,LOC150935	2q37.3	rs59626274	240500249	intergenic	C	T	0.248	0.002	-1.34	5.78E-14	10.18	0.0679	0.078
LINC01237	2q37.3	rs78458145	242877035	ncRNA_intronic	G	A	0.285	0.007	-1.41	3.14E-15	11.27	0.8416	0.085
SPCS3,VEGFC	4q34.2	rs17688188	177471059	intergenic	G	A	0.222	0.030	-1.31	5.99E-13	9.29	0.9999	0.072
TCF24	8q13.1	rs112859779	67860369	G141S	C	T	0.216	2.02E-04	-1.69	3.33E-16	12.12	1.0000	0.090
CCDC171,C9orf92	9p22.3	rs12350582	16135142	intergenic	A	G	0.255	0.841	0.89	1.49E-08	5.44	3.34E-10	0.046
LINGO2	9p21.1	rs56942085	28518911	intronic	G	A	0.061	1	1.81	3.90E-08	5.07	1.42E-10	0.044
TMOD1	9q22.33	rs1052270	100275424	intronic	C	T	0.344	0.042	-0.89	2.71E-08	5.21	1.97E-10	0.045
MIR3134	9q31.3	rs79461840	114822158	ncRNA_intronic	T	C	0.076	0.003	1.93	1.40E-08	5.46	3.51E-10	0.046
LOC101928775,LINC00474	9q33.1	rs4979583	118571428	intergenic	C	T	0.325	0.874	0.90	4.61E-09	5.88	9.29E-10	0.049
OLFML2A	9q33.3	rs79134766	127561723	A208T	G	A	0.219	0.054	-1.97	1.70E-19	14.92	1.0000	0.107
MYEOV,LINC01488	11q13.3	rs76855873	69154455	intergenic	C	T	0.269	0.023	-1.11	1.23E-11	8.15	1.89E-13	0.064
ARHGAP32	11q24.3	rs371331393	128839272	stopgain	G	A	0.171	1	3.77	9.32E-27	20.88	1.0000	0.143
CD163L1	12p13.31	rs138525217	7528192	splicing	C	T	0.161	1	4.33	6.20E-23	17.77	1.0000	0.125
SLC2A14	12p13.31	rs118107419	7980394	intronic	C	A	0.262	0.012	-1.40	1.44E-14	10.71	8.60E-08	0.081
DRAM1,CCDC53	12q23.2	rs7964241	102338235	intergenic	A	G	0.278	0.166	0.91	1.95E-08	5.33	3.58E-13	0.045
DRAM1,CCDC53	12q23.2	rs56168082	102339295	intergenic	G	A	0.276	0.166	0.89	3.93E-08	5.07	1.97E-13	0.044
CUL4A,LAMP1	13q34	rs74115822	113931854	intergenic	G	A	0.112	0.001	1.83	1.12E-10	7.32	0.9960	0.058
MIR365A,PARN	16p13.12	rs11646803	14463359	intergenic	C	T	0.376	0.074	-0.79	4.76E-10	6.77	0.9422	0.055
LOC102724084	16q23.2	rs75861150	80193070	ncRNA_intronic	T	C	0.091	2.17E-04	-1.93	1.27E-08	5.51	0.0520	0.046
SCARF1	17p13.3	17_1538628	1538628	E639E	G	C	0.265	0.147	-0.94	6.02E-09	5.79	0.0008	0.048

MINK1	17p13.2	rs72835045	4780795	intronic	G	A	0.220	1.16E-06	-1.38	1.69E-12	8.92	0.9984	0.069
SLC47A1	17p11.2	rs2440154	19444987	intronic	G	A	0.204	0.791	0.98	1.42E-08	5.47	0.0004	0.046
SLC47A1	17p11.2	rs2289668	19454640	intronic	C	T	0.204	0.791	0.98	1.43E-08	5.46	0.0004	0.046
NAPA-AS1	19q13.32	rs55800589	47990889	ncRNA_intronic	G	C	0.364	2.37E-06	-0.96	3.35E-13	9.53	1.0000	0.073
DSCAM	21q22.2	rs727333	41702947	intronic	C	A	0.257	4.75E-04	-1.36	3.69E-14	10.38	0.1247	0.077
LRRC3	21q22.3	rs116969723	45876716	P63P	G	A	0.233	0.014	-1.45	3.83E-15	11.23	0.8753	0.083
RFPL2,SLC5A4	22q12.3	rs117398778	32609178	intergenic	T	C	0.138	1.56E-05	1.32	2.00E-09	6.24	0.9397	0.051

GWAS, genome-wide association study; HWE p, Hardy-Weinberg equilibrium (HWE) p-value; log10BF, log10 transformed Bayes factor; lnOR, natural log-transformed odds ratio; PIP, posterior probability; MAF, minor allele frequency.

[a] major/minor allele type.

[b] lnOR and *P*-value were estimated by IA GWAS.

[c] PIP, log10BF, and heritability (h^2) of individual variants were estimated via FINEMAP program to identify causal susceptibility to IA.

Supplementary Table S2. Expression of four causal candidate genes in 13 brain tissues, 4 blood vessels (3 artery tissues and EBV cells), and whole blood (transcripts per millions, TPM)

Tissue or cell types, TPM values	*GBA*	*TCF24*	*OLFML2A*	*ARHGAP32*
Artery-Aorta	26.6381	0.0000	2.3707	4.8536
Artery-Coronary	22.6014	0.0000	8.8402	4.3448
Artery-Tibial	18.5048	0.0000	9.5401	3.8799
Cells-EBV-transformedlymphocytes	13.2542	0.0000	0.4718	0.0667
Brain-Amygdala	6.4943	0.0758	1.0522	9.2092
Brain-Anteriorcingulatecortex(BA24)	8.5819	0.0529	0.9995	13.5215
Brain-Caudate(basalganglia)	9.8782	0.0782	1.2439	12.8529
Brain-CerebellarHemisphere	9.8881	0.0000	0.7039	4.8586
Brain-Cerebellum	9.5836	0.0166	1.0395	7.3319
Brain-Cortex	10.6011	0.0549	1.4575	19.6950
Brain-FrontalCortex(BA9)	13.1018	0.0508	1.1425	23.0975
Brain-Hippocampus	6.9035	0.0895	1.1693	6.8002
Brain-Hypothalamus	10.5743	0.1027	1.1759	6.1858
Brain-Nucleusaccumbens(basalganglia)	10.6235	0.0644	0.7168	12.7685
Brain-Putamen(basalganglia)	7.9459	0.0384	1.2849	9.1537
Brain-Spinalcord(cervicalc-1)	13.7758	0.1448	1.2204	4.5931
Brain-Substantianigra	8.7868	0.0567	1.3471	5.0176
WholeBlood	33.1310	0.0000	0.0980	0.2274

Supplementary Table S3. Associations of 29 SNPs in the previous GWAS (Hong et al. 2019) and replication stages.

Gene	Chr	SNP	M	m	MAF	HWE p	Stage 1[a]		Stage 2[b]		Stage 3[c]	
							lnOR[e]	*P*[e]	ln(OR)[e]	*P*[e]	ln(OR)[d]	*P*[d]
PRDM2	1p36.21	rs61775135	C	A	0.285	0.626	-1.15	3.59E-13	4.00	0.7495	NA	NA
GBA	1q22	rs75822236	C	T	0.166	1	5.08	1.09E-19	NA	NA	NA	NA
FMO4	1q24.3	rs3737926	C	T	0.264	0.079	-1.01	1.83E-10	0.24	0.7801	NA	NA
RNF144A	2p25.1	rs6741819	C	T	0.247	0.022	-1.38	4.05E-14	-1.57	0.06768	NA	NA
HDAC4, LOC150935	2q37.3	rs59626274	C	T	0.248	0.002	-1.34	5.78E-14	1.17	0.4364	-0.59	0.2636
LINC01237	2q37.3	rs78458145	G	A	0.285	0.007	-1.41	3.14E-15	-1.13	0.09543	NA	NA
SPCS3,VEGFC	4q34.2	rs17688188	G	A	0.222	0.03	-1.31	5.99E-13	1.33	0.04135	NA	NA
TCF24	8q13.1	rs112859779	C	T	0.216	2.02E-04	-1.69	3.33E-16	0.19	0.8588	NA	NA
CCDC171, C9orf92	9p22.3	rs12350582	A	G	0.255	0.841	0.89	1.49E-08	-0.01	0.9763	0.31	0.1932
LINGO2	9p21.1	rs56942085	G	A	0.061	1	1.81	3.90E-08	0.86	0.2593	0.78	0.03301
TMOD1	9q22.33	rs1052270	C	T	0.344	0.042	-0.89	2.71E-08	-0.76	0.107	-0.47	0.05375
MIR3134	9q31.3	rs79461840	T	C	0.076	0.003	1.93	1.40E-08	2.17	0.00245	NA	NA
LOC101928775, LINC00474	9q33.1	rs4979583	C	T	0.325	0.874	0.9	4.61E-09	0.78	0.03896	NA	NA
OLFML2A	9q33.3	9_127561723	G	A	0.219	0.054	-1.97	1.70E-19	NA	NA	NA	NA
MYEOV, LINC01488	11q13.3	rs76855873	C	T	0.269	0.023	-1.11	1.23E-11	0.76	0.308	NA	NA
ARHGAP32	11q24.3	rs371331393	G	A	0.171	1	3.77	9.32E-27	NA	NA	NA	NA
CD163L1	12p13.31	rs138525217	C	T	0.161	1	4.33	6.20E-23	NA	NA	NA	NA
SLC2A14	12p13.31	rs118107419	C	A	0.262	0.012	-1.4	1.44E-14	0.58	0.5324	0.60	0.05648
DRAM1, CCDC53	12q23.2	rs7964241	A	G	0.278	0.166	0.91	1.95E-08	0.95	0.0465	0.32	0.1598
CUL4A, LAMP1	13q34	rs74115822	G	A	0.112	0.001	1.83	1.12E-10	-1.80	0.154	NA	NA
MIR365A, PARN	16p13.12	rs11646803	C	T	0.376	0.074	-0.79	4.76E-10	-0.02	0.971	NA	NA
LOC102724084	16q23.2	rs75861150	T	C	0.091	2.17E-04	-1.93	1.27E-08	-16.60	0.9906	NA	NA
SCARF1	17p13.3	17_1538628	G	C	0.265	0.147	-0.94	6.02E-09	1.16	0.1904	1.10	1.17E-04
MINK1	17p13.2	rs72835045	G	A	0.22	1.16E-06	-1.38	1.69E-12	1.83	0.1472	NA	NA

SLC47A1	17p11.2	rs2440154	G	A	0.204	0.791	0.98	1.42E-08	0.97	0.03906	0.56	0.01104
NAPA-AS1	19q13.32	rs55800589	G	C	0.364	2.37E-06	-0.96	3.35E-13	NA	NA	NA	NA
DSCAM	21q22.2	rs727333	C	A	0.257	4.75E-04	-1.36	3.69E-14	-0.39	0.5288	NA	NA
LRRC3	21q22.3	rs116969723	G	A	0.233	0.014	-1.45	3.83E-15	-1.11	0.4892	NA	NA
RFPL2, SLC5A4	22q12.3	rs117398778	T	C	0.138	1.56E-05	1.32	2.00E-09	1.69	0.01276	NA	NA

HWE p, Hardy-Weinberg equilibrium p-value; M, major allele; m, minor allele; MAF, minor allele frequency.

[a] The estimation of stage 1 has been reported in the previous GWAS composed of 250 patients with IA and 296 controls (Hong et al. 2019 J Clin Med).

[b] Stage 2 shows replication results of 29 genome-wide significant signals in a hospital-based independent study composed of 50 patients with IA and 46 controls.

[c] Stage 3 shows replication results of 29 genome-wide significant signals in an independent study composed 50 hospital-based patients and 575 healthy controls without underlying diseases. Controls were extracted from 8,105 subjects the Rural and Mid-size City cohort in Korean Genome Epidemiology Study.

[d] Natural log-transformed odds ratio (ln(OR)) and p-values were estimated by multivariate logistic regression analysis adjusting for gender, age, hypertension, diabetes mellitus, hyperlipidemia, smoking status, and four principal component values.
